# Data Analysis and Visualization of Newspaper Articles on Thirdhand Smoke: A Topic Modeling Approach

**DOI:** 10.2196/12414

**Published:** 2019-01-29

**Authors:** Qian Liu, Qiuyi Chen, Jiayi Shen, Huailiang Wu, Yimeng Sun, Wai-Kit Ming

**Affiliations:** 1 School of Journalism and Communication Jinan University Guangzhou China; 2 National Media Experimental Teaching Demonstration Center Jinan University Guangzhou China; 3 School of Medicine Jinan University Guangzhou China; 4 Department of Obstetrics & Gynaecology The First Affiliated Hospital, Sun Yat-sen University Guangzhou China; 5 Harvard Medical School Harvard University Boston, MA United States; 6 Faculty of Medicine The Chinese University of Hong Kong Shatin, NT China (Hong Kong); 7 Li Ka Shing Faculty of Medicine University of Hong Kong Pokfulam China (Hong Kong)

**Keywords:** media concerns, topic modeling, third-hand smoke, tobacco, indoor air quality

## Abstract

**Background:**

Thirdhand smoke has been a growing topic for years in China. Thirdhand smoke (THS) consists of residual tobacco smoke pollutants that remain on surfaces and in dust. These pollutants are re-emitted as a gas or react with oxidants and other compounds in the environment to yield secondary pollutants.

**Objective:**

Collecting media reports on THS from major media outlets and analyzing this subject using topic modeling can facilitate a better understanding of the role that the media plays in communicating this health issue to the public.

**Methods:**

The data were retrieved from the Wiser and Factiva news databases. A preliminary investigation focused on articles dated between January 1, 2013, and December 31, 2017. Use of Latent Dirichlet Allocation yielded the top 10 topics about THS. The use of the modified LDAvis tool enabled an overall view of the topic model, which visualizes different topics as circles. Multidimensional scaling was used to represent the intertopic distances on a two-dimensional plane.

**Results:**

We found 745 articles dated between January 1, 2013, and December 31, 2017. The United States ranked first in terms of publications (152 articles on THS from 2013-2017). We found 279 news reports about THS from the Chinese media over the same period and 363 news reports from the United States. Given our analysis of the percentage of news related to THS in China, Topic 1 (Cancer) was the most popular among the topics and was mentioned in 31.9% of all news stories. Topic 2 (Control of quitting smoking) was related to roughly 15% of news items on THS.

**Conclusions:**

Data analysis and the visualization of news articles can generate useful information. Our study shows that topic modeling can offer insights into understanding news reports related to THS. This analysis of media trends indicated that related diseases, air and particulate matter (PM_2.5_), and control and restrictions are the major concerns of the Chinese media reporting on THS. The Chinese press still needs to consider fuller reports on THS based on scientific evidence and with less focus on sensational headlines. We recommend that additional studies be conducted related to sentiment analysis of news data to verify and measure the influence of THS-related topics.

## Introduction

Thirdhand smoke (THS) is an important public health issue and has been an increasingly popular topic for decades in China since its first mention in 2009 [1]. Aggregating media reports from major media outlets and analyzing the coverage using topic modeling may help shed light on the role that the media plays in communicating this health concept. Thirdhand smoke consists of residual tobacco smoke pollutants that remain on surfaces and in dust after tobacco has been smoked. These pollutants are re-emitted into the gas phase (ie, off-gassing) or react with oxidants and other compounds in the environment to yield secondary pollutants [2]. Evidence supports the presence of THS in indoor environments. Thirdhand smoke is found in enclosed spaces where habitual smoking occurs, such as residences and automobiles [3]. This phenomenon is associated with health hazards: research has shown that residual nicotine from tobacco smoke absorbed onto indoor surfaces reacts with ambient nitrous acid to form carcinogenic tobacco-specific nitrosamines, which can cause significant levels of DNA damage in human cell lines [3]. The related acute and long-term risks of THS include disease and premature mortality. Children and infants are particularly susceptible to THS exposure [4].

Given the increasing interest in THS, the mass media has focused on delivering and communicating information on this topic to public audiences. Both in China and abroad, there have been media reports related to THS. According to search results of news articles mentioning THS, the United States ranks first in terms of topic mentions. However, previous studies have shown that fewer people are aware of the harms of THS than that of secondhand smoke [5]. Because the mass media is a key player in communicating health-related information, it could play a positive role in helping the public understand the risks of THS and the ways to protect themselves from it [6]. We therefore decided to compare this topic between China and the United States.

Multimodal data modeling combines information from different resources. Topic modeling refers to statistical models in which unstructured data are structured in accordance with latent themes to deal with multimodal data. Latent Dirichlet Allocation (LDA) is the most popular form of topic modeling and is a generative probabilistic modeling method for converting visual words into images and visual word documents [7-9]. Topic modeling has broad applications in various fields such as text mining [10], medicine [11-13], economics [14], and social network analysis [15]. To the best of our knowledge, however, there have been very few studies using LDA to evaluate the media’s treatment of THS. As a result, we used LDA modeling method for our analysis despite its’ being a common method. This paper aims to determine the current patterns and the role of mass communication related to THS.

## Methods

### Data Collection

We used the Wiser database for Chinese news content (from the Wise News website) and the Factiva database (from the Dow Jones website) to retrieve the international news articles. The Wiser database is an ever-growing, professional Chinese media content database that contains more than one million data entries. It is currently the best source for Chinese media content given its large volume of data and timely updates. Factiva is an international news and information database that includes nearly 33,000 premium sources such as licensed publications, influential websites, blogs, images, and videos. To get a general idea of the topic of THS, we conducted a preliminary investigation of the Factiva database and retrieved articles dated between January 1, 2013, and December 31, 2017. The Wiser database includes data published only within the last five years. However, many of the articles we flagged merely noted the topic of smoking without further elaborating on the issue of THS. We next narrowed down the entries to only those newspaper sources pertaining to THS; we believe that articles from newspapers are much more reliable than those from other sources. We used LDA to further analyze the Chinese news articles.

An LDA topic model is a model with a three-level hierarchical Bayesian model. The basic assumption of this model is a combination of words belonging to different topics [16]. LDA suggests that there may be multiple topics in an article and that the wording in that article or paper reflects the exact set of topics that the reporter wished to address. Using Gibbs Sampling techniques, a method that estimates the marginal distribution of interested variables, we can determine the topics among the data pool [17].

### Processing

**Figure 1:**
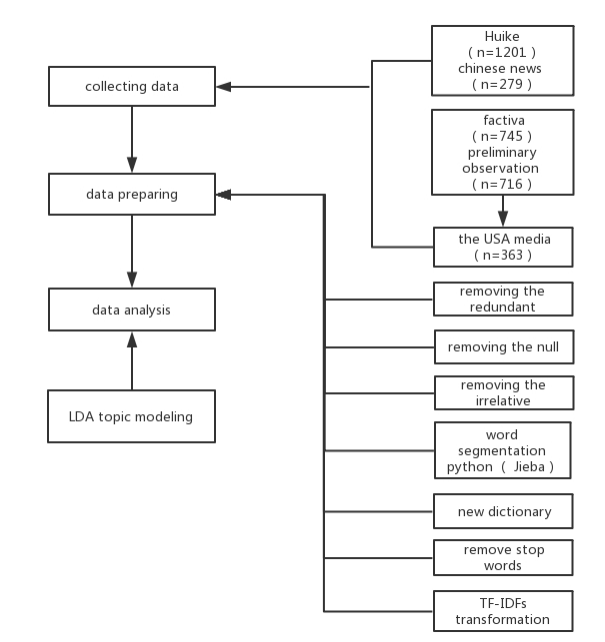
Data processing chart. LDA: Latent Dirichlet Allocation; TF-IDF: frequency–inverse document frequency.

Data processing (Figure 1) was conducted before applying LDA modeling by using Python to do data cleaning and the Python package, Jieba, to do the segmentation [18,19]. First, the redundant and null data were chosen to be removed, followed by removal of irrelevant information such as advertisements. Next, word segmentation was conducted using the Jieba package. However, THS articles with a lot of terminology were calculated and added based on the calculation of the words’ information entropy and term frequency to avoid the influence of unprofessional dictionaries. New terms such as “China Anticancer Association,” “Family Doctor,” “Shenzhen Municipal Government,” “Air Circulation,” “Air Purification,” “Chinese Preventive Medicine Association,” “Tobacco Monopoly,” “Tobacco Market,” and so on, were added to the dictionary for further analysis. Furthermore, common Chinese stop words were removed such as “a,” “of”, “it,” etc. Next, a document-term matrix was built, and term frequency–inverse document frequency (TF-IDF) was used in the data processing.

Multiple studies have been conducted related to the choice of LDA topic number and the explanations of each topic. In previous research related to topic number, 10-30 topics were assigned nearly the same log-likelihood measure. Therefore, we adopted the number 10 for the topic parameter. By analyzing key words, topic content was generated accordingly. If we considered only the statistical measures, the results might not be interpretable by humans [20]. Therefore, by combining both statistical measures and manual interpretation, we selected 10 topics to analyze by using Python version 3.6.1 with the LDAvis tool [16]. We set λ=1 and recovered 10 topics and their keywords (Table 1). To elaborate on the topics, the topic name was generated based on the given keywords (Table 1) as well.

**Figure 2:**
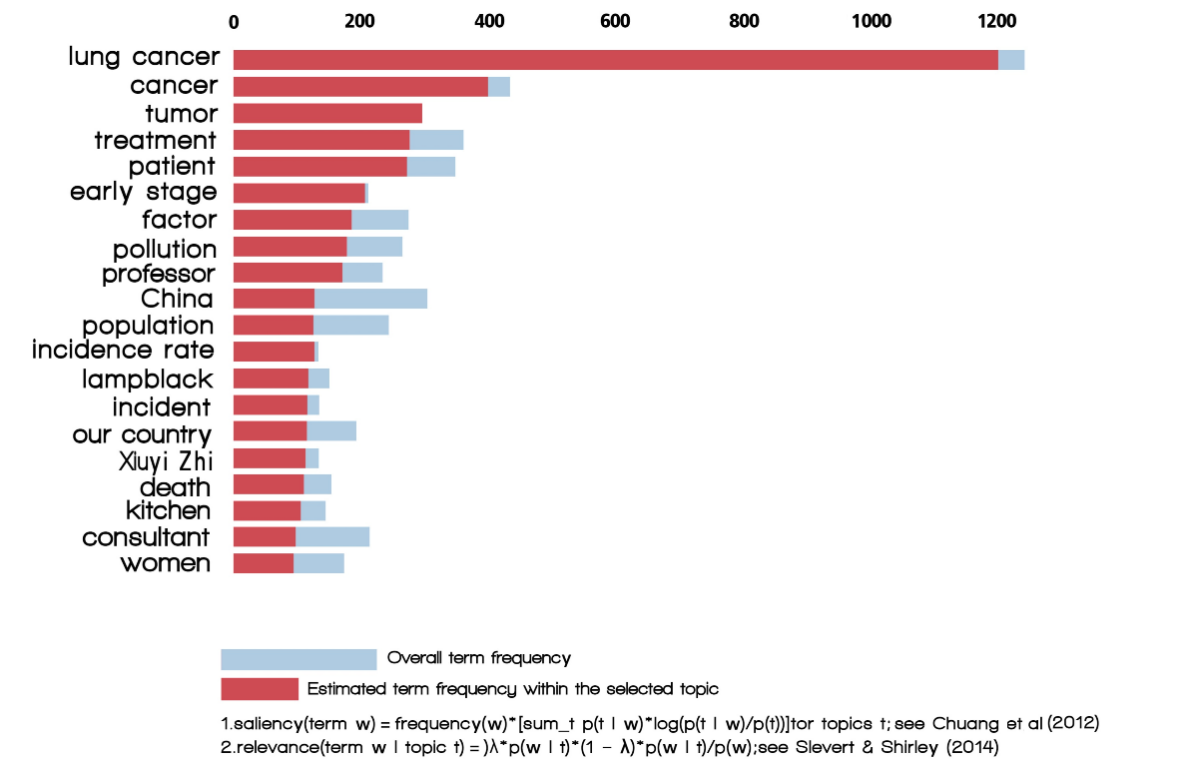
Intertopic distance map.

**Figure 3:**
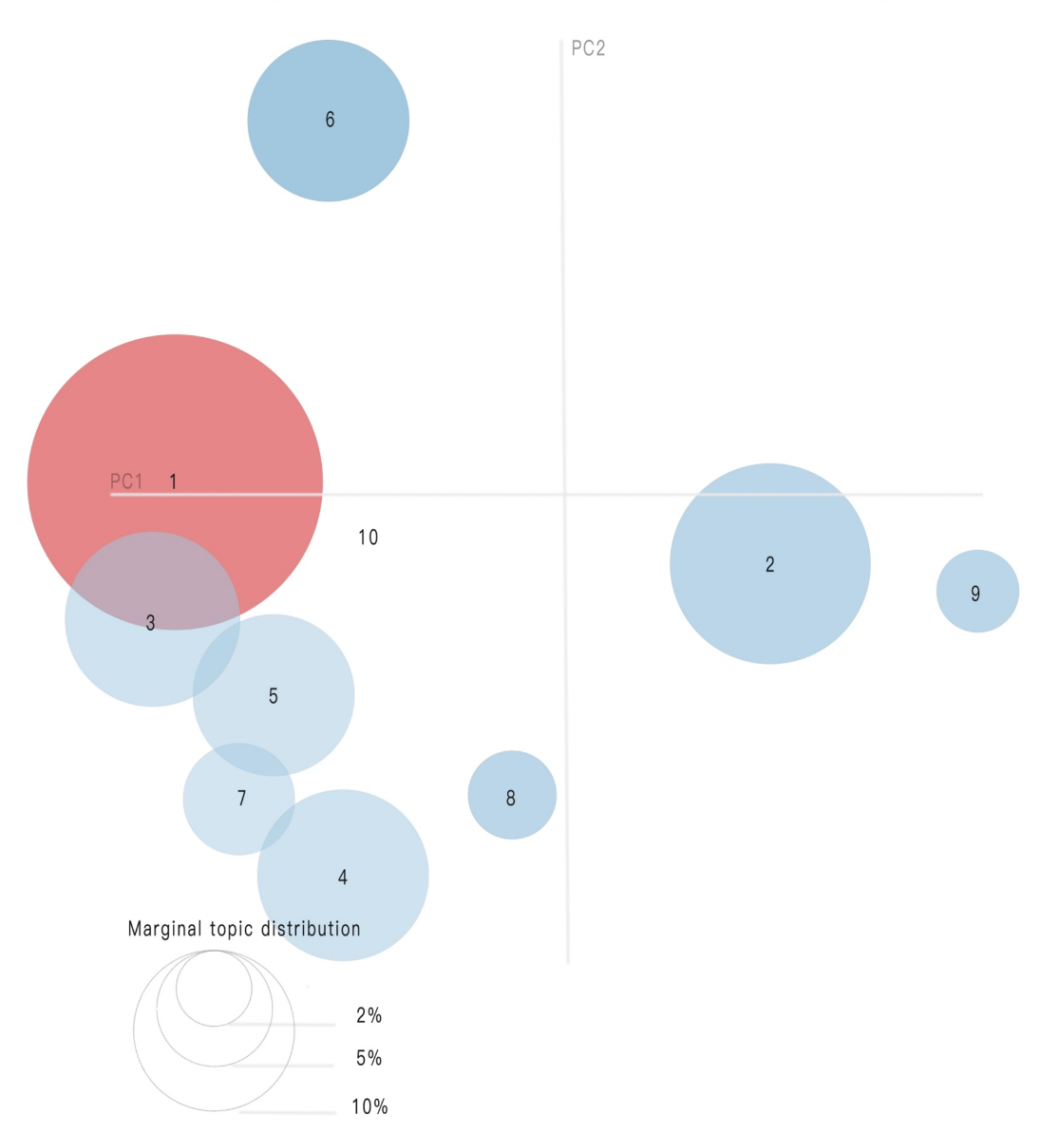
Top 20 most relevant terms for topic 1.

We sought topics that overlapped as in the visualization shown in Figures 2 and 3. In this two-dimensional plane, topics are represented as cycles whose centers are determined by the computed topics’ distances [16]. We categorized the topics into three main primary groups: links to related diseases, air and particulate matter (PM_2.5_), and control and restrictions (Table 1).

**Table 1:** Topic classification and keywords.

Classification group and topic name		Key words	Percentage (%)
**Related diseases**			
	Topic 1: cancer	Lung cancer, cancer, tumor, treatment, patient, risk factor, pollution, professor, China, population, prevalence, diseases pattern, prevention of air pollution	31.9
	Topic 5: risks of smoking	Smoker, movement, body, nicotine, content, quitting smoking, experts, symptom related to dead smokers	10.1
	Topic 7: diseases induced by smoking	Asthma, citizen, hospital, doctor, patient, treatment, smoker, time, long-term, breath, chairman, cause	4.9
	Topic 3: susceptible population	Children, research, food, contact, cause, influence, environment, increase, body, clothes, smog, content, reveal, professor, indoor, smoker, female	11.4
	Topic 4: quitting smoking	Quit smoking, smoker, smoke, hospital, drug, breath, doctor, work, smoker, content, one kind, introduction, treatment, chairman	11.1
	Topic 8: relevant research	Introduction, reveal, cigarette, children, smoke, officer, smoker, increase, factor, place, reason, environment, relevant	3.2
**Air and PM_2.5_**			
	Topic 6: air quality	PM_2.5_, indoor, concentration, severe, microgram, air, pollution, smog, influence, kitchen, cooking fume	9.7
**Control and restrictions**			
	Topic 9: classic smoking control case	Shenzhen, tobacco control, activity, citizen, place, rule, investigation, smoker, work, over, condition, patients, quit smoking, control, indoor, public place, increase	2.6
	Topic 10: public control	Public place, quit smoking, ban tobacco, rule, place, professor children, indoor, China, tobacco control, body, influence, body, female worker, reveal	0.1
	Topic 2: control of quitting smoking	Quit smoking, ban tobacco, third-hand smoke, public place, place, rule, ban, indoor, control, work, country, smoke, society, China, Beijing, relevant, smoker, outdoor	15

## Results

We found 745 articles dated between January 1, 2013, and December 31, 2017. After excluding repeated articles, 716 news stories remained. This study collected 1201 news articles via the Wiser database platform. After culling repeated news and irrelevant entries, we recovered 288 news stories by selecting publications from only the largest media outlets in China representing the most influential mass media outlets. Finally, after the data-cleaning process was complete, we amassed 279 articles dated from January 1, 2013, through December 31, 2017. A comparison of the Chinese news and American news is listed in Table 2. Worldwide attention has been focused on THS [1,21], and the top five regions of origin of these articles and their corresponding prevalence are listed in Table 3. Not surprisingly, the United States ranks first, publishing roughly 152 articles on THS from 2013-2017. There has been an obvious decrease in interest in THS from the Chinese media within the last 5 years.

Figure 2 presents an overall view of the topic model. We plotted different topics as circles, where overall prevalence was calculated as the areas of the circles. The centers of each topic were determined by computing the distance between topics; we used multidimensional scaling to represent the intertopic distances on a two-dimensional plane [22]. PC1 indicates the transverse axis and PC2 indicates the longitudinal axis in Figure 2.

Figure 3 shows a bar chart in a descending order of the top 20 most useful terms, for interpreting a topic. The overlaid bars represent a given term’s corpus-wide frequency and the topic-specific frequency, as noted previously in the literature [23].

**Figure 4:**
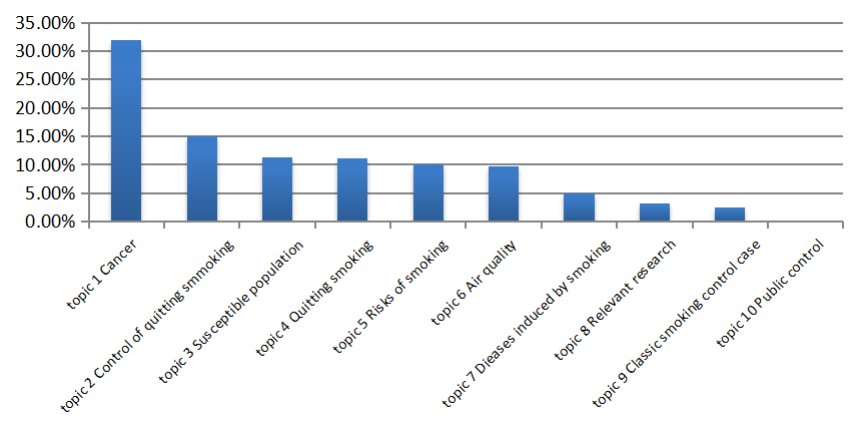
Topic percentage allocation.

Figure 4 shows the percentage of news related to THS in China. Topic 1 (Cancer) was the most popular. Roughly 32% of all news stories noted this topic. Topic 2 (Control of quitting smoking) was included in approximately 15% news of THS-related news. Topic 3 (Susceptible population), Topic 4 (Quitting smoking), Topic 5 (Risks of smoking), and Topic 6 (Air quality) were each involved in roughly 10% of news stories. There were no news reports related to Topic 10.

Our results show that the Chinese press was less concerned with THS than the American press. Relative to 2013-2015, the number of reports in 2016 mentioning THS declined slightly. This trend indicates that the popularity of the topic might follow a worldwide trend (Table 3). We also observed a trend of increasing concern after a Chinese American scientist presented important findings about THS, linking THS to possible DNA damage that can cause diseases such as cancer [24,25]. This finding was widely reported both in China and the United States. It had an influence, particularly in government policy making (eg, legislation on smoke-free environments) [26]. Thus, there is a link between academic concerns and mass media concerns in China and the United States [5].

We also analyzed the three classifications, including links to diseases category, air and the PM_2.5_ category, and the control and restrictions category (Table 1 and Figure 4). The first category, links to diseases, appeared in 72.6% of articles. This finding suggests that this category was frequently reported in China. Some health information can be misleading when delivered via the media, and new media platforms enable the rapid spread of news, some of it emotional or personal. This can include information that is false or misleading. As a result, the public still has a limited or even inaccurate understanding of THS. This situation was consistent with the findings of a prior study [27].

Roughly one tenth (9.7%) of the all-news stories were related to the air and PM_2.5_ category. The media seemed to focus on sensational topics related to THS, even if some links were tenuous. Therefore, professionalism and credibility in reporting are of vital importance. Furthermore, the control and restrictions category was included in 17.1% of all stories. This category is a unique one. While the Chinese media emphasizes control and restrictions, the American media focuses on conveying to smokers that they should quit the habit.

In comparing the Chinese and American news, we found an enormous variation between reports on the same topic in terms of the following three aspects. The Chinese news focused on children and the elderly as victims, whereas the American news focused more on pregnant women. The Chinese media discussed a number of measures and restrictions the government or authorities took or should be taken related to smoking, while the American press focused more on how individuals could eliminate risks. Furthermore, Chinese news articles were, on average, shorter than American news articles.

**Table 2:** Chinese and American news about thirdhand smoke.

Year	Chinese news articles (n)	American news articles (n)
2013	78	52
2014	57	114
2015	74	54
2016	29	38
2017	41	105
Total	279	363

**Table 3:** Countries publishing news about thirdhand smoke.

Country	Articles (n)
United States	152
United Kingdom	56
Canada	37
India	33
Australia	21

## Discussion

### Principal Findings

Thirdhand smoke has been a growing topic for years in China. Topic modeling offers insights into understanding news reports related to THS. This study collected media reports from the United States and China and analyzed them using topic modeling. Specifically, we retrieved Chinese reports about THS from major newspaper and press outlets in China and compared this information with English-language news on the same subject from the United States. Our results revealed that the American press was more concerned with THS than the Chinese. In the Chinese media, three major concerns emerged: links of THS to disease, air and PM_2.5_, and tobacco control and restrictions.

Our results indicate that the media has served a public health function by publishing reports that warn the general public about THS-related dangers such as lung cancer, asthma, tumors, and other diseases linked to tobacco. Furthermore, these articles emphasize the possible risks of THS and the susceptible populations (eg, children) in indoor environments. Smoking cessation and relevant research are also reported. Therefore, the media do communicate the risks associated with smoking, as well as information about prevention and smoking cessation.

For air quality and PM_2.5_-related reports, the media attempts to connect sensational concepts with the topic, even if such concepts are not quite related. Therefore, professionalism and credibility are of vital importance. In addition, new media platforms enable the rapid spread of news that is more emotional or personal, or possibly false, which is a serious concern. Aided by new media platforms such as WeChat, Weibo, and Jinri Toutiao (the biggest new media platforms in China), information that may include misleading or exaggerated concepts can quickly be disseminated on personalized newsfeeds.

In terms of topics related to tobacco control and restrictions, the Chinese media emphasizes control and restrictions more than the United States. The American media focuses on helping smokers quit the habit.

### Strengths and Limitations

Topic modeling is a new method that reveals the major topics in media reports and singles out several key concerns and findings related to the topics. Data analysis and the visualization of news articles can generate useful information. However, there is a limitation to be noted in our study. We included only major media databases, which might omit some news content from new media, such as WeChat posts. Therefore, we may have missed some news stories.

### Conclusion

Thirdhand smoke is an important public health issue. Collecting media reports on THS from major media outlets and analyzing this subject using topic modeling can facilitate a better understanding of the role that the media plays in communicating this health issue to the public. We conclude that the Chinese press still needs to consider fuller reports on THS rather than simply reporting sensational headlines and needs to show more professionalism by not publishing articles that lack scientific evidence. We recommend that additional studies be conducted related to sentiment analysis of news data to verify and measure the influence of topics revealed from the reports. For example, scientists could measure the educational function of the media for public health or study the influence of misleading information about THS generated by news reports.

## Abbreviations

LDA: Latent Dirichlet Allocation
TF-IDF: frequency–inverse document frequency
THS: thirdhand smoke
PM: particulate matter
